# The Frequency-Response Electroretinogram Distinguishes Cone and Abnormal Rod Function in *rd12* Mice

**DOI:** 10.1371/journal.pone.0117570

**Published:** 2015-02-23

**Authors:** Xufeng Dai, Hua Zhang, Ying He, Yan Qi, Bo Chang, Ji-jing Pang

**Affiliations:** 1 Eye Hospital, School of Ophthalmology and Optometry, Wenzhou Medical University, Wenzhou, China; 2 Department of Ophthalmology, College of Medicine, University of Florida, Gainesville, Florida, United States of America; 3 The Jackson Laboratory, Bar Harbor, Maine, United States of America; University Zürich, SWITZERLAND

## Abstract

Early studies on *Rpe65* knockout mice reported that remaining visual function was attributable to cone function. However, this finding has been challenged more and more as time has passed. Electroretinograms (ERGs) showed that *rd12* mice, a spontaneous animal model of RPE65 Leber’s congenital amaurosis, had sizeable photopic responses. Unfortunately, the recorded ERG waveform was difficult to interpret because of a remarkably delayed peak-time, which resembles a rod response more than a cone response. Here, we compare flicker ERGs in animals with normal rod and cone function (C57BL/6J mice), pure rod function *(cpfl5 mice*), and pure cone function *(Rho^-/-^* mice) under different adaptation levels and stimulus intensities. These responses were then compared with those obtained from *rd12* mice. Our results showed that normal rods respond to low frequency flicker (5 and 15 Hz) and that normal cones respond to both low and high frequency flicker (5–35 Hz). As was seen in *cpfl5 mice*, *rd12* mice had recordable responses to low frequency flicker (5 and 15Hz), but not to high frequency flicker (25 and 35 Hz). We hypothesize that abnormal rods may be the source of residual vision in *rd12* mice, which is proved correct here with double mutant *rd12*mice. In this study, we show, for the first time, that frequency-response ERGs can effectively distinguish cone- and rod-driven responses in the *rd12* mouse. It is another simple and valid method for evaluating the respective contributions of retinal rods and cones.

## Introduction

Leber’s congenital amaurosis type 2 (LCA2) is caused by retinal pigment epithelium-specific 65 kDa protein (RPE65) mutations, which lead to early onset of blindness [1–4]. Expressed inside of RPE cells, RPE65 is necessary for regeneration of 11-*cis*-retinal, the ligand of rod and cone opsins [5]. As an isomerohydrolase of the visual cycle, RPE65 allows the phototransduction cascade to begin by providing the chromophore that captures photon energy. Therefore, it helps to maintain normal visual function [6]. Rhodopsin in rods is composed of opsin, an apoprotein opsin, and 11-*cis* retinal, a chromophore. Similarly, cone pigment is composed of cone opsin and 11-*cis* retinal [7]. Hence, loss of RPE65 function may result in loss of both rod and cone photoreceptor function [6, 8, 9].

Early studies of RPE65 deficient animals [6, 10] and LCA patients [11] have concluded that residual visual function is attributable to cones. However, this finding has been challenged. There is also evidence that shows that cones, along with rods, rapidly degenerate as *Rpe65* ^*-/-*^ mice age [12]. In 2001, Seeliger et al. [13] first showed that residual electroretinogram (ERG) signals disappeared when rod-related rhodopsin was knocked out in *Rpe65* ^*-/-*^ mice. Subsequently, Cachafeiro et al. [14] used the optomotor test to demonstrate that in *Rpe65* ^*-/-*^ mice, rod function mimics cone function, in part through vision mediation under photopic conditions.

Discovery of the *rd12* mouse, a mouse that spontaneously developed a similar RPE65 mutation as LCA patients, has made a powerful, new, independent model available for studying LCA2 pathogenesis and progression [15]. It should be noted that the *rd12* mouse is not identical to previously available *Rpe65* ^*-/-*^ knockout mice because the knockout mouse had no fundus abnormalities. In contrast, *rd12* mice develop small white dots throughout their retinas. By examining retinal function, it was determined that cone, but not rod, photoreceptors rapidly degenerate in the *rd12* mouse [16]. In contrast to common rapid photoreceptor degenerations [17–19], residual photopic ERGs remain stable for at least the first five months of life in *rd12* mice. The recorded waveform was confusing in that it had a markedly delayed peak-time, which is generally representative of rod, not cone, function. Based on reports by Seeliger et al. [13] and Cachafeiro et al. [14], the standard rod-suppressive background (30 cd/m^2^) may be ineffective in *rd12* mice.

Flicker ERGs can also be used to differentiate rod and cone function. Critical flicker fusion frequency (CFF) is the highest frequency at any light intensity that an observer can resolve flicker. This parameter is used to compare temporal resolution capabilities between rod and cone photoreceptors [20–22]. High-frequency flicker can be used to tell rod function from cone function because cones have a higher CFF than rods. In this study, we used three mouse models, in addition *rd12* mice, to explore the frequency response of rods and cones. The C57BL/6J mouse has normal rod and cone function, the *cpfl5* mouse (*Cnga3* mutation) only has functioning rods [23], and the rhodopsin knockout (Rho-^*/-*^) mouse has only functioning cones [24].

Here, we explore frequency-response ERGs in various mouse models to differentiate cone- and abnormal rod-driven responses in a spontaneous LCA2 mouse model. To confirm our results, this was also done in *rd12* mice with further induced mutations (double-mutant) in *cpfl5* [23] or *Rho* ^*−/−*^ [24].

## Materials and Methods

### Animals

Three to 5 week old wild type C57BL/6J, *cpfl5*, *Rho* ^*-/-*^, and *rd12* mice were obtained from the Jackson Laboratory (Bar Harbor, ME), bred, and maintained in the animal facilities of University of Florida (Gainesville, FL) and Wenzhou Medical University (Wenzhou, China). We generated two types of double mutant mice by crossbreeding *rd12* mice with *cpfl5* or Rho-^*/-*^ mice and subsequently breeding each F1 heterozygote. Double mutant mice were identified in the F2 generation by polymerase chain reaction (PCR) analysis of genomic DNA, as described for the individual lines [6, 25, 26]. All mice had free access to food and water and were maintained in a 12-hour light/12-hour dark cycle with an ambient light intensity of 15 lux. Five mice from each strain underwent testing between 3 and 5 weeks of age. All experiments were approved by both the University of Florida and the Wenzhou Medical University institutional animal care and use committee (Permit Number: wydw2014–0072) and were conducted in accordance with the ARVO Statement for the Use of Animals in Ophthalmic and Vision Research.

### Animal Preparation

After overnight dark-adaptation, mice were anesthetized with a solution of ketamine (70mg/kg) and xylazine (5mg/kg) under dim red light. Pupils were dilated with 1% atropine sulfate. After instillation of a topical corneal anesthetic (0.5% proparacaine hydrochloride), 1.0% methylcellulose gel was applied to the eyes to prevent corneal dehydration and to allow for better electrical contact with the recording electrode. Specially made contact lens electrodes (Hansen Ophthalmics, Iowa City, IA) were placed over the corneas to record the ERGs. Needle reference and ground electrodes were inserted into the cheek and tail, respectively [27]. Full field ERGs were recorded using a custom-built ganzfeld and a computer-based system (Roland Consult, Wiesbaden, Germany and LKC Technologies, Gaithersburg, MD). White light emitting diodes (LEDs, 450–780nm) were used as stimulation and background light sources. All LED intensities *were* measured *by* a digital illuminometer (*Shenzhen Oway Technology*, *Shenzhen*, *China)*. The band pass of the amplifiers was 1–500Hz.

### Electroretinograms

Scotopic ERGs were recorded at-1.85 log cd-s/m^2^ and 0 log cd-s/m^2^ stimulus intensity [23] with an interstimulus interval of 30 seconds. The final ERG waveform was the average of five individual waveforms. This was done to increase the signal:noise ratio. Photopic ERGs were recorded after mice were exposed to a steady background illumination of 30 cd/m^2^ for 10 minutes. The ERGs were evoked by +0.65 log cd-s/m^2^, and 50 individual ERG waveforms, recorded every 0.4 seconds, were averaged to obtain the final ERG waveform [23]. The ganzfeld stimulus used was a white, full-field flash with a duration of 2 ms and a color temperature of 7000K.

### Flicker Electroretinograms

Scotopic and photopic ERGs were recorded in mice at flicker rates of 5, 15, 25, and 35 Hz. While mice were dark-adapted, dim stimuli (-1.85 log cd-s/m^2^) only activated rods and bright stimuli (0 log cd-s/m^2^) activated both rods and cones [23]. Photopic ERGs were elicited at +0.65 log cd-s/m^2^ after a steady background illumination of 30 cd/m^2^ was presented for 10 minutes [28, 29]. The amplitudes and peak times of flicker ERG responses were evaluated. To verify reproducibility, flicker ERGs were recorded using two different systems (Roland Consult, Wiesbaden, Germany and LKC Technologies, Gaithersburg, MD) and the same recording protocol.

### Statistical Analyses

All statistical analyses were performed with SPSS statistical software (ver. 18.0, IBM Corporation, Armonk, NY). Because of the limited sample size, frequency-response ERG data in the four mice strains were analyzed using a non-parametric test. This included a Kruskal-Wallis ANOVA on rank, followed by a multiple comparison procedure (Dunnett´s test). Statistical significance was defined as *P* < 0.05.

## Results

### Electroretinograms of Wild Type C57BL/6J, cpfl5, Rho^−/−^, and rd12 Mice

**Fig 1:**
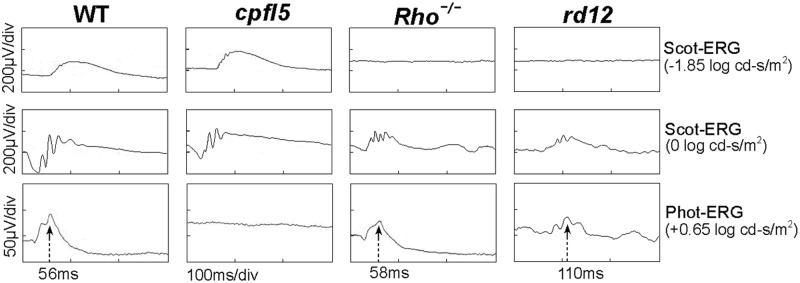
Scotopic and photopic electroretinograms from 1 month old *rd12*, wild type, *cpfl5*, and *Rho* ^*−/−*^ mice. Under light-adaptation, an electroretinogram signal was detectable in the *rd12* mouse, but had a markedly delayed peak-time (110 ms) compared to wild type (56ms) and *Rho* ^*−/−*^ (58ms) mice.

Scotopic ERGs showed that retinas of wild type C57BL/6J and *cpfl5* mice, but not Rho-^*/-*^ and *rd12* mice, had sensitive rod responses (Fig. 1). Under light-adapted conditions and with bright stimuli, sizeable ERG signals were recorded from *rd12* eyes, but had obvious peak-time delays compared to cone responses in C57BL/6J and Rho-^*/-*^ mice (Fig. 1). Significant differences were found in b-wave peak times among *rd12*, C57BL/6J, and Rho-^*/-*^ mice (114.0 ± 15.5, 57.4 ± 5.9, and 58.2 ± 3.8 ms, respectively, *P* < 0.001). The multiple comparison procedure (Dunnett´s test) revealed significant differences between *rd12* and wild type mice (n = 5, *P* < 0.001) and between *rd12* and Rho-^*/-*^ mice (n = 5, *P* < 0.001).

### Flicker Electroretinograms in Four Mice Strains at Frequencies of 5, 15, 25, and 35 Hz

All ERGs were recorded with two different ERG-systems using the same experimental settings and conditions. The ERG amplitudes and peak times from the same mice were not significantly different when recorded with the different systems under the same stimulus and background intensity (n = 5, *P* > 0.05).

**Fig 2:**
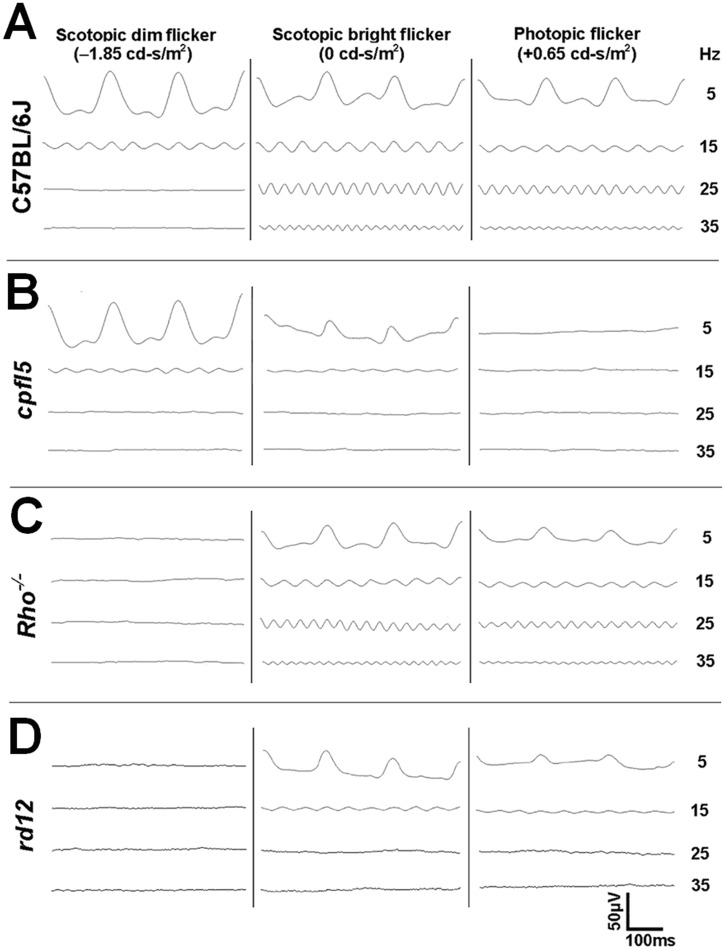
Scotopic and photopic flicker electroretinograms from 1 month old wild type, *cpfl5*, *Rho* ^*−/−*^, and *rd12* mice. A scotopic dim stimulus (-1.85 log cd-s/m^2^) activated rods and a bright stimulus (0 log cd-s/m^2^) activated both rods and cones. Photopic stimuli (+0.65 log cd-s/m^2^) activated cones and desensitized, abnormal rods. Under dark- and light-adaptation, the wild type mouse (A) responded to both low (5 and 15 Hz) and high (25 and 35 Hz) flicker frequencies. The *cpfl5* mouse (B) only responded to low frequency (5 and 15Hz) flicker under dark-adaptation. Under dark- and light-adaptation, the *Rho* ^*−/−*^ mouse (C) responded to both low (5 and 15 Hz) and high (25 and 35 Hz) flicker frequencies, but the *rd12* mouse (D) only responded to low frequency (5 and 15 Hz) flicker.

**Fig 3:**
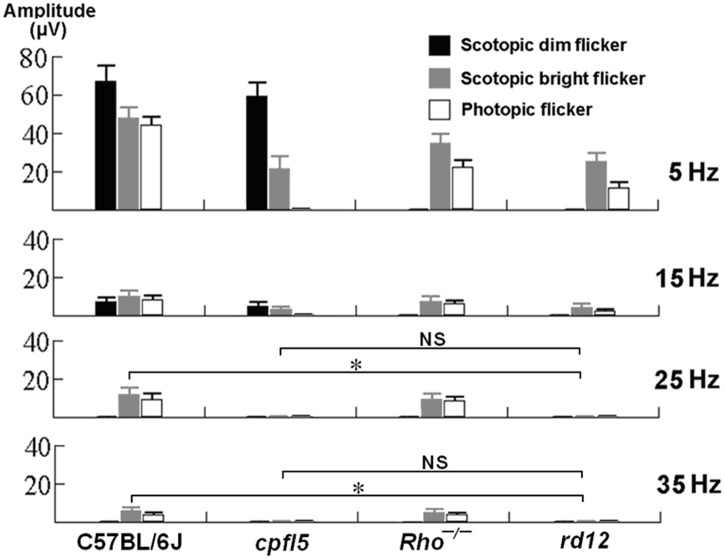
Amplitudes of frequency-response electroretinograms in 1 month old wild type, *cpfl5*, *Rho* ^*−/−*^, and *rd12* mice. Under dark-adaptation, stimulus intensity for dim and bright flicker were-1.85 log cd-s/m^2^ and 0 log cd-s/m^2^, respectively. Photopic responses were elicited with a +0.65 log cd-s/m^2^ stimulus. Flicker ERG amplitudes were evaluated. Columns and bars represent mean ± standard deviation (n = 5 mice). *indicates *P* < 0.001, NS = no statistical difference.

The C57BL/6J mice have normal rod and cone photoreceptor function. A dim scotopic stimulus (-1.85 log cd-s/m^2^) only activates rods and a bright stimulus (0 log cd-s/m^2^) activates both rods and cones. Dark-adapted flicker ERGs in C57BL/6J mice were detectable with dim light stimuli presented at a low frequency (5 and 15 Hz), but not with the same stimuli presented at a high frequency (25 and 35 Hz, Figs. 2A and 3). With bright stimuli, dark-adapted flicker ERGs were observed at all four frequencies examined (Figs 2A and 3). Cone-driven light-adapted flicker ERGs elicited by bright stimuli were confirmed with stimuli ranging between 5 and 35 Hz (Figs. 2A and 3).

In *cpfl5* mice (pure rod function), dark-adapted flicker ERGs were detectable with dim stimuli presented at a low frequency (5 and 15 Hz), but not at a high frequency (25 and 35 Hz) (Figs. 2B and 3). When bright stimuli were presented, flicker responses were also detectable, but only for low frequency stimuli (Figs. 2B and 3). No light-adapted flicker responses were observed at any of the four stimulus frequencies examined.

In Rho-^*/-*^ mice (pure cone function), dark-adapted flicker responses were not observed when dim stimuli were presented at any of the four frequencies tested. When bright stimuli were presented, both dark- and light-adapted flicker ERGs were observed at all frequencies examined (Figs. 2C and 3).

In *rd12* mice, the dim light stimuli did not elicit dark-adapted flicker responses at any of the four frequencies tested. The bright stimuli elicited dark-adapted flicker responses with a stimulus frequency of 5 and 15 Hz, but not of 25 and 35 Hz (Figs. 2D and 3 and Table 1). Light-adapted flicker ERGs were detectable when the stimulus frequency was 5 and 15 Hz, but not when it was 25 and 35 Hz (Figs. 2D and 3 and Table 1). As in photopic 5 Hz results, the 15 Hz flicker ERG had a delayed peak time in eyes of *rd12* mice (80.4 ± 5.2 ms, n = 5 mice) compared to eyes of wild type mice (53.2 ± 3.0 ms, n = 5 mice, *P* < 0.001).

**Table 1:** Scotopic and photopic ERG amplitudes **in the four mice strains examined.**

		Mouse model				*P* Value		
Amplitude, μV		C57BL/6J	*cpfl5*	Rho-^*/-*^	*rd12*	*rd12* vs. C57BL/6J	*rd12* vs. *Rho* ^*-/-*^	*rd12* vs. *cpfl5*
Scotopic ERG (0 log cd-s/m^2^)	25Hz	12.2 ± 4.0	1.0 ± 0.2	10.0 ± 2.8	0.8 ± 0.4	<0.001	<0.001	>0.05
	35Hz	5.6 ± 1.5	0.5 ± 0.4	4.8 ± 1.6	0.8 ± 0.2	<0.001	<0.001	>0.05
Photopic ERG (+0.65 log cd-s/m^2^)	25Hz	9.4 ± 2.8	0.6 ± 0.2	8.6 ± 2.0	0.4 ± 0.2	<0.001	<0.001	>0.05
	35Hz	3.8 ± 1.2	0.8 ± 0.2	4.2 ± 1.0	0.6 ± 0.4	<0.001	<0.001	>0.05

Flicker frequencies of 25 and 35 Hz are presented.

Data presented as mean ± standard deviation. n = 5 mice for each mouse model examined. *P* values calculated using a multiple comparison procedure (Dunnett´s test).

**Fig 4:**
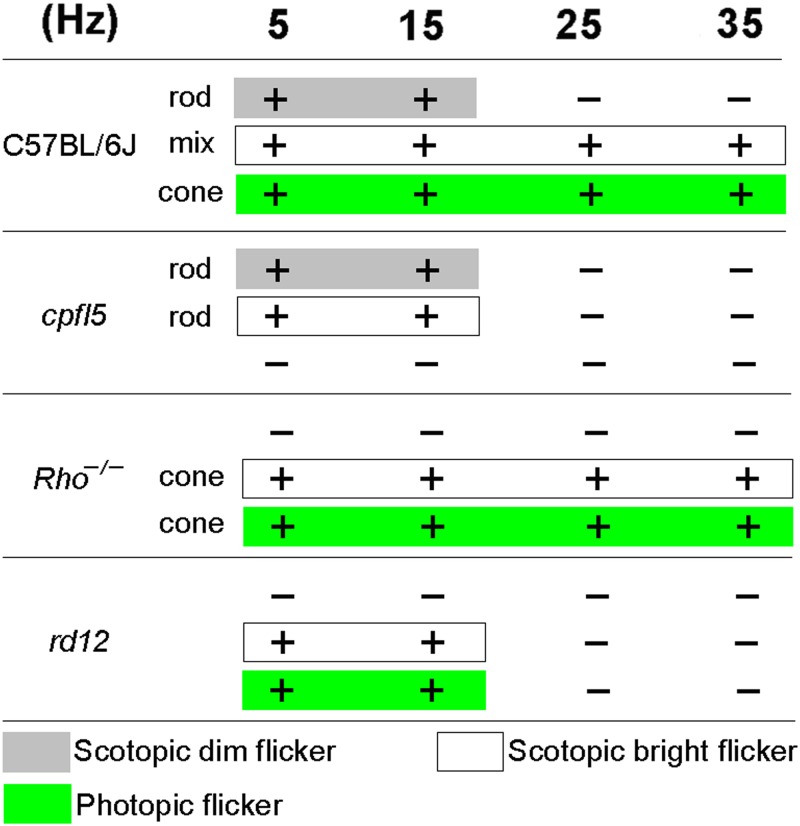
Comparison of flicker electroretinograms in all mice strains examined under different adaptation and stimulus intensities. The C57BL/6J mice have normal rod and cone function, the *cpfl5* mice have pure rod function, and the Rho-^*/-*^ mice have pure cone function. Rods respond to low frequency (5 and 15Hz) flicker and cones respond to both low (5 and 15 Hz) and high frequency (25 and 35 Hz) flicker. The *rd12* mice only responded to low-frequency flicker. + indicates that flicker ERGs existed,—indicates that flicker ERGs did not exist, 5–35 Hz = flicker frequency.

Comparing and contrasting flicker ERGs in the four strains of mice (Fig. 4) revealed that *rd12* mice (*Rpe65* natural deficiency) lost their high frequency flicker response, which resembled rod-driven responses observed in *cpfl5* mice and scotopic (rod) flicker ERGs in C57BL/6J mice.

### Electroretinograms for C57BL/6J, Rpe65 Single Mutant, and Double Mutant rd12 Mice

**Fig 5:**
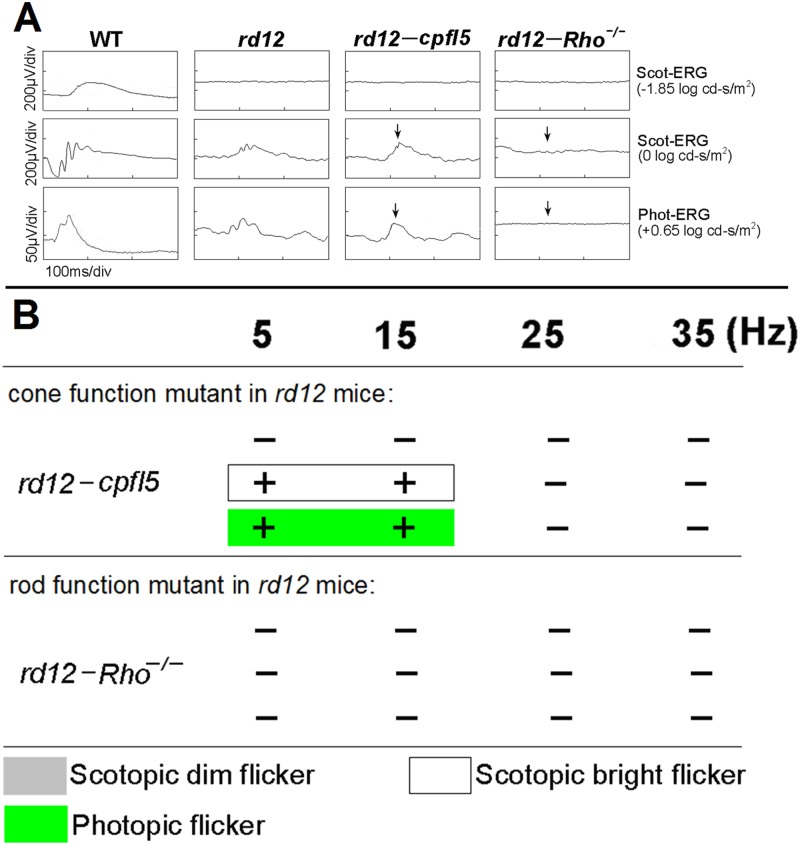
Comparison of electroretinograms between *rd12-cpfl5* and *rd12-Rho* ^*-/-*^ double-mutant mice. Electroretinogram (ERG, A) and flicker ERG (B) responses of the *rd12* mouse (*Rpe65* single mutant) was compared with those of the double-mutant *rd12* mouse generated with *cpfl5* (cone photoreceptor function loss 5) or Rho^*-/-*^ (rhodopsin knockout) animals. The ERGs of the *rd12* and *rd12-cpfl5* mouse were similar, but no discernible responses were observed in the *rd12*-Rho-^*/-*^ mouse. + indicates flicker ERGs existed,—indicates flicker ERGs did not exist, 5–35 Hz = flicker frequency.

As a reminder, *cpfl5* mice have pure rod function and Rho-^*/-*^ mice have pure cone function. Cone and rod function was selectively impaired in *rd12* mice by generating *rd12-cpfl5* and *rd12-Rho* ^*-/-*^ double-mutant mice, respectively. The ERGs of *rd12-cpfl5* mice were comparable with ERGs of *rd12* mice. However, *rd12-*Rho-^*/-*^ mice failed to show any substantial ERG in response to bright stimuli (Fig. 5).

## Discussion

Some investigators believe that using photopic conditions alone may not provide entirely pure cone responses because remaining rods may still retain some level of responsiveness [13, 14]. To avoid the influence of rod responsiveness, we examined responses to flicker frequencies above the temporal resolution of rods because cone photoreceptors are capable of responding to higher CFFs than rods [21, 22]. Our previous work showed that the maximum flicker frequency rods can respond to is 19–23 Hz. However, cones can respond to flicker with frequencies higher than 35 Hz (data not shown). Therefore, we examined responses to stimuli flickering at 15 and 25 Hz as a first step in effectively differentiating cone- and rod-driven flicker responses. To better understand frequency-response features, flicker frequencies of 5 and 35 Hz were also examined (10 Hz intervals). Flicker ERGs revealed that response amplitude was related to flicker frequency. Low frequency flicker stimuli generally elicit relatively larger waves in rods and cones. Near the CFF, responses were smaller. First, we examined flicker ERGs of normal C57BL/6J mice and determined differences between rod- and cone-driven responses. Dark-adapted dim stimuli were set at-1.85 log cd-s/m^2^, which was below the threshold of cones [23]. Thus, scotopic flicker ERGs elicited with low intensity light only reflected rod-driven function. We found that, under our experimental conditions, normal rods respond to flicker at frequencies no higher than 15 Hz. Bright stimuli were set at 0 log cd-s/m^2^, which is capable of exciting both rods and cones [23]. We were able to record responses at both low and high flicker frequencies during dark-adaptation. After 10 minutes of light-adaptation, normal rod function of C57BL/6J mice was fully suppressed. Cone-driven flicker ERGs were observed at frequencies between 5 and 35 Hz. This finding is typical of cone frequency-responses. Furthermore, we examined cone deficient *cpfl5* [23] and rod deficient Rho-^*/-*^ [24] mice to confirm the frequency-response features of rods and cones. We found that Rho-^*/-*^ mice (purely cone function) respond to both low and high flicker frequencies. However, *cpfl5* mice (purely rod function) did not respond to high frequency stimuli under dark-adaptation (Fig. 4).

Our results showed that rods can respond to flicker in the low frequency range (5 and 15 Hz), but that cones can respond to both low and high frequencies (5–35 Hz). Because *rd12* mice responded to low frequency stimuli (5 and 15Hz), but not high frequency stimuli (25 and 35 Hz), we hypothesize that the abnormal rod system in this spontaneous RPE65-LCA model may be the source of residual vision. To confirm this hypothesis, we selectively impaired either rod or cone function in *rd12* mice by generating *rd12-cpfl5* and *rd12*-Rho^*-/-*^ mice. If rods are truly responsible for light responses in *rd12* mice, similar ERGs would be obtainable from *rd12* and *rd12-cpfl5* mice. Alternatively, if the cone system is responsible, ERGs from *rd12* mice would resemble those of *rd12*-Rho-^*/-*^ mice. We found that ERGs of *rd12* and *rd12-cpfl5* mice were similar, and that no detectable responses were observed in *rd12*-Rho-^*/-*^ mice (Fig. 5).

Flicker ERGs of *rd12* mice were similar to rod-driven responses observed in *cpfl5* mice, except that rods had a decreased sensitivity to both flashes and background illumination (Fig. 4). In *Rpe65* ^*-/-*^ mice, the retina had a sensitivity deficit of about 3–4 orders of magnitude relative to normal rods. The dramatically reduced light sensitivity is believed to result from rhodopsin (or similar product) levels that are several orders of magnitude lower than normal [13, 30]. This may explain why the standard rod-suppressive background (30 cd/m^2^) was ineffective in *rd12* mice. The ERG is a field potential that reflects the electrical response to a light stimulus [31]. In the *rd12* mouse with a RPE65 protein defect, signals generated by photoreceptors were then transmitted to bipolar cells, which lead to abnormal peak times and amplitudes of the positive ERG components.

In this study, we have shown, for the first time, that frequency-response ERGs can effectively distinguish cone- and desensitized, abnormal rod-driven responses. Our method offers a simple and practical way to evaluate the respective contributions of the retinal rod and cone systems.

## References

[1] Gu SM, Thompson DA, Srikumari CR, Lorenz B, Finckh U, et al (1997) Mutations in *RPE65* cause autosomal recessive childhood-onset severe retinal dystrophy. Nat Genet 17(2):194–197. 9326941 10.1038/ng1097-194

[2] Morimura H, Fishman GA, Grover SA, Fulton AB, Berson EL, et al (1998) Mutations in the RPE65 gene in patients with autosomal recessive retinitis pigmentosa or Leber congenital amaurosis. Proc Natl Acad Sci U S A 95(6):3088–3093. 9501220 10.1073/pnas.95.6.3088 PMC19699

[3] Porto FB, Perrault I, Hicks D, Rozet JM, Hanoteau N, et al (2002) Prenatal human ocular degeneration occurs in Leber's congenital amaurosis (LCA2). J Gene Med 4(4):390–396. 12124981 10.1002/jgm.278

[4] Testa F, Maguire AM, Rossi S, Pierce EA, Melillo P, et al (2013) Three-year follow-up after unilateral subretinal delivery of adeno-associated virus in patients with Leber congenital Amaurosis type 2. Ophthalmology 120(6):1283–1291. 10.1016/j.ophtha.2012.11.048 23474247 PMC3674112

[5] Moiseyev G, Chen Y, Takahashi Y, Wu BX, Ma JX (2005) RPE65 is the isomerohydrolase in the retinoid visual cycle. Proc Natl Acad Sci U S A 102(35):12413–12418. 16116091 10.1073/pnas.0503460102 PMC1194921

[6] Redmond TM, Yu S, Lee E, Bok D, Hamasaki D, et al (1998) *Rpe65* is necessary for production of 11-*cis*-vitamin A in the retinal visual cycle. Nat Genet 20(4):344–351. 9843205 10.1038/3813

[7] Rohrer B, Lohr HR, Humphries P, Redmond TM, Seeliger MW, et al (2005) Cone opsin mislocalization in *Rpe65* ^-/-^ mice: a defect that can be corrected by 11-*cis* retinal. Invest Ophthalmol Vis Sci 46(10):3876–3882. 16186377 10.1167/iovs.05-0533

[8] Jacobson SG, Aleman TS, Cideciyan AV, Sumaroka A, Schwartz SB, et al (2005) Identifying photoreceptors in blind eyes caused by RPE65 mutations: prerequisite for human gene therapy success. Proc Natl Acad Sci U S A 102(17):6177–6182. 15837919 10.1073/pnas.0500646102 PMC1087926

[9] Samardzija M, Tanimoto N, Kostic C, Beck S, Oberhauser V, et al (2009) In conditions of limited chromophore supply rods entrap 11-*cis*-retinal leading to loss of cone function and cell death. Human Molecular Genetics 18(7):1266–1275. 10.1093/hmg/ddp026 19147682

[10] Veske A, Nilsson SE, Narfström K, Gal A (1999) Retinal dystrophy of Swedish Briard/Briard-beagle dogs is due to a 4-bp deletion in RPE65. Genomics 57(1):57–61. 10191083 10.1006/geno.1999.5754

[11] Lorenz B, Gyürüs P, Preising M, Bremser D, Gu S, et al (2000) Early-onset severe rod-cone dystrophy in young children with RPE65 mutations. Invest Ophthalmol Vis Sci 41(9):2735–2742. 10937591

[12] Ekesten B, Gouras P, Salchow DJ (2001) Ultraviolet and middle wavelength sensitive cone responses in the electroretinogram (ERG) of normal and Rpe65 -/- mice. Vision Res 41(19):2425–2433. 11483174 10.1016/s0042-6989(01)00140-7

[13] Seeliger MW, Grimm C, Ståhlberg F, Friedburg C, Jaissle G, et al (2001) New views on RPE65 deficiency: the rod system is the source of vision in a mouse model of Leber congenital amaurosis. Nat Genet 29(1):70–74. 11528395 10.1038/ng712

[14] Cachafeiro M, Bemelmans AP, Canola K, Pignat V, Crippa SV, et al (2010) Remaining rod activity mediates visual behavior in adult *Rpe65* ^-/-^ mice. Invest Ophthalmol Vis Sci 51(12):6835–6842. 10.1167/iovs.09-3870 20702833

[15] Pang JJ, Chang B, Hawes NL, Hurd RE, Davisson MT, et al (2005) Retinal degeneration 12 (*rd12*): a new, spontaneously arising mouse model for human Leber congenital amaurosis (LCA). Mol Vis 11: 152–162. 15765048

[16] Pang J, Boye SE, Lei B, Boye SL, Everhart D, et al (2010) Self-complementary AAV-mediated gene therapy restores cone function and prevents cone degeneration in two models of Rpe65 deficiency. Gene Ther 17(7):815–826. 10.1038/gt.2010.29 20237510 PMC3014850

[17] Takahashi M, Miyoshi H, Verma IM, Gage FH (1999) Rescue from photoreceptor degeneration in the *rd* mouse by human immunodeficiency virus vector-mediated gene transfer. J Virol 73(9):7812–7816. 10438872 10.1128/jvi.73.9.7812-7816.1999 PMC104309

[18] Pang JJ, Dai X, Boye SE, Barone I, Boye SL, et al (2011) Long-term Retinal Function and Structure Rescue Using Capsid Mutant AAV8 Vector in the *rd10* Mouse, a Model of Recessive Retinitis Pigmentosa. Mol Ther 19(2):234–242. 10.1038/mt.2010.273 21139570 PMC3034861

[19] Dai X, Han J, Qi Y, Zhang H, Xiang L, et al (2014) AAV-Mediated Lysophosphatidylcholine Acyltransferase 1 (*Lpcat1*) Gene Replacement Therapy Rescues Retinal Degeneration in *rd11* Mice. Invest Ophthalmol Vis Sci 55(3):1724–1734. 10.1167/iovs.13-13654 24557352 PMC3968931

[20] Nowak LM, Green DG (1983) Flicker fusion characteristics of rod photoreceptors in the toad. Vision Res 23(9):845–849. 6415914 10.1016/0042-6989(83)90051-2

[21] Pearson P, Timney B (1999) Differential effects of alcohol on rod and cone temporal processing. J Stud Alcohol 60(6):879–883. 10606502 10.15288/jsa.1999.60.879

[22] Rubin GR, Kraft TW (2007) Flicker assessment of rod and cone function in a model of retinal degeneration. Doc Ophthalmol 115(3):165–172. 17674067 10.1007/s10633-007-9066-9

[23] Pang JJ, Deng WT, Dai X, Lei B, Everhart D, et al (2012) AAV-mediated cone rescue in a naturally occurring mouse model of CNGA3-achromatopsia. PLoS One 7(4):e35250 10.1371/journal.pone.0035250 22509403 PMC3324465

[24] Jaissle GB, May CA, Reinhard J, Kohler K, Fauser S, et al (2001) Evaluation of the rhodopsin knockout mouse as a model of pure cone function. Invest Ophthalmol Vis Sci 42(2):506–513. 11157890

[25] Biel M, Seeliger M, Pfeifer A, Kohler K, Gerstner A, et al (1999) Selective loss of cone function in mice lacking the cyclic nucleotide-gated channel CNG3. Proc Natl Acad Sci U S A 96(13):7553–7557. 10377453 10.1073/pnas.96.13.7553 PMC22124

[26] Humphries MM, Rancourt D, Farrar GJ, Kenna P, Hazel M, et al (1997) Retinopathy induced in mice by targeted disruption of the rhodopsin gene. Nat Genet 15(2):216–219. 9020854 10.1038/ng0297-216

[27] Li W, Kong F, Li X, Dai X, Liu X, et al (2009) Gene therapy following subretinal AAV5 vector delivery is not affected by a previous intravitreal AAV5 vector administration in the partner eye. Mol Vis 15: 267–275. 19190735 PMC2633462

[28] Lei B, Yao G, Zhang K, Hofeldt KJ, Chang B (2006) Study of rod- and cone-driven oscillatory potentials in mice. Invest Ophthalmol Vis Sci 47(6):2732–2738. 16723493 10.1167/iovs.05-1461

[29] Deng WT, Dinculescu A, Li Q, Boye SL, Li J, et al (2012) Tyrosine-mutant AAV8 delivery of human MERTK provides long-term retinal preservation in RCS rats. Invest Ophthalmol Vis Sci 53(4):1895–1904. 10.1167/iovs.11-8831 22408006 PMC3995567

[30] Fan J, Rohrer B, Moiseyev G, Ma JX, Crouch RK (2003) Isorhodopsin rather than rhodopsin mediates rod function in RPE65 knock-out mice. Proc Natl Acad Sci U S A 100(23):13662–13667. 14578454 10.1073/pnas.2234461100 PMC263870

[31] Pardue MT, Peachey NS (2014) Mouse b-wave mutants. Doc Ophthalmol 128(2):77–89. 10.1007/s10633-013-9424-8 24395437 PMC6010232

